# Supplementary material for: Content-rich biological network constructed by mining PubMed abstracts
Source: BMC Bioinformatics. 2004 Oct 8;5:147. doi: 10.1186/1471-2105-5-147 (PMC528731; doi:10.1186/1471-2105-5-147)
Supplement: Additional File 2 — The original results of the above study (non-essential files are deleted to keep the file size under the limit set by BMC bioinformatics). [file 1471-2105-5-147-S2.bz2 › chilibotAdditionalFile2/dip10/355ID8120391E4997/html/IL10_IL10RA.html]

 


 **IL10** and **IL10RA** 
  
Found 91 abstracts in PubMed, retrieved 10.  
 

 What does Google say? 
 PDF only 
| .edu only 

---

**Interactive relationship** (e.g. stimulation, inhibition, etc)

**Neutral relationship**- IL 10  [ **IL10** ]  signaling via IL 10E1 is dependent on tyrosine phosphorylation in the IL 10R  [ **IL10RA** ]  alpha chain in human primary prostate cancer cell lines.  Ref: 12802285 Oncogene, 2003

**Non-interactive relationship** (e.g. studied together, co-existance, homology, etc.)

- However, because anti IL 10R  [ **IL10RA** ]  also released IFN gamma independent effects, IL 10  [ **IL10** ]  appears to act more broadly and suppresses multiple antileishmanial mechanisms.  Ref: 12870129 J Infect Dis, 2003
- As judged by IL 10R  [ **IL10RA** ]  blockade, endogenous IL 10  [ **IL10** ]  primarily regulates killing in L. donovani infection by suppressing production of and responses to the Th1 cell type cytokines, IL 12, and IFN gamma.  Ref: 12870129 J Infect Dis, 2003
- In established Leishmania donovani visceral infection in normal mice, anti interleukin IL 10  [ **IL10** ]  receptor IL 10R  [ **IL10RA** ]  monoclonal antibody MAb treatment induced intracellular parasite killing within liver macrophages.  Ref: 12870129 J Infect Dis, 2003
- IL 10R  [ **IL10RA** ]  expression did not correlate with plasma IL 10  [ **IL10** ]  levels or disease activity.  Ref: 12673894 Clin Exp Rheumatol< MedlineTA>Clin Exp Rheumatol, 2003
- OBJECTIVE We aimed to determine the expression of the interleukin 10  [ **IL10** ]  receptor IL10R  [ **IL10RA** ]  on circulating leukocytes in SLE and rheumatoid arthritis, and correlate this with plasma IL 10  [ **IL10** ]  levels and disease activity.  Ref: 12673894 Clin Exp Rheumatol< MedlineTA>Clin Exp Rheumatol, 2003
- During relapse, IL 10  [ **IL10** ] , IL 10R1  [ **IL10RA** ] , ICAM 1, VCAM 1 mRNA levels and neurological scores show positive correlations.  Ref: 12620647 J Neuroimmunol, 2003
- To investigate the role of other loci in the susceptibility to RA, we have performed an analysis of several polymorphisms in genes of immune related function IL 10  [ **IL10** ]  108 1 92 promoter single nucleotide polymorphisms SNPs, IL 10G and IL 10R  [ **IL10RA** ]  microsatellites, IL 6 622 promoter SNP, FcgammaRIIIA Val Phe 158 polymorphism, IL 1 receptor antagonist VNTR, and the IKBL 738 T C mutation.  Ref: 12618859 Genes Immun, 2003
- In a rat model of chronic relapsing EAE, expression levels of IL 10  [ **IL10** ]  and its receptor IL 10R1  [ **IL10RA** ] , ICAM 1 and VCAM 1 mRNA in the spinal cord are markedly increased.  Ref: 12620647 J Neuroimmunol, 2003
- Immune suppression was mediated independently of IL 10  [ **IL10** ]  and TGF beta production, as neutralizing antibodies for IL 10  [ **IL10** ] , IL 10R  [ **IL10RA** ] , and TGF beta were unable to revert suppression, and IL 10  [ **IL10** ]  deficient CD4 T cells were able to mediate in vitro and in vivo suppression.  Ref: 12609834 Blood, 2003
- In IL 10R1  [ **IL10RA** ]  G330R expressing monocytes, the inhibitory effect of IL 10  [ **IL10** ]  on TNF alpha production was diminished, indicating that this variant may be a loss of function allele.  Ref: 12759436 J Immunol, 2003
- Structural analysis of the S138G variant revealed that the substitution of S138G may interfere with binding of IL 10  [ **IL10** ]  to IL 10R1  [ **IL10RA** ] .  Ref: 12759436 J Immunol, 2003
